# Beauty is only skin deep: An examination of physical attractiveness, attractive personality, and personal grooming on criminal justice outcomes

**DOI:** 10.1371/journal.pone.0291922

**Published:** 2023-10-11

**Authors:** Krysta N. Knox, Michael F. TenEyck

**Affiliations:** 1 School of Criminal Justice, University of Cincinnati, Cincinnati, Ohio, United States of America; 2 Department of Criminology and Criminal Justice, The University of Texas at Arlington, Arlington, Texas, United States of America; University of North Carolina at Chapel Hill, UNITED STATES

## Abstract

Research has found that physical attractiveness is related to a host of benefits across life outcomes. Within the field of criminal justice, physical attractiveness appears to afford individuals leniency at various decision points. This research, however, often fails to include measures of personal presentation beyond simply measuring physical attractiveness. The current study extends this line of research by investigating how physical attractiveness, personality attractiveness, and grooming are related to criminal justice processing decisions. Results from negative binomial and logistic regression analyses indicate that when assessed alone, physical attractiveness significantly decreased the odds of criminal behavior by 18 percent, arrest by 11 percent, conviction by 14 percent, and incarceration by 28 percent—controlling for race, age, and gender. When controlling for personality and grooming, however, physical attractiveness results in a 47 to 53 percent increase in the risk of experiencing these outcomes while having an attractive personality and being well-groomed significantly reduced such risk. These results highlight the importance of considering other factors of appearance and personal presentation when considering how attractiveness influences criminal justice outcomes.

## Introduction

It has been said that ‘beauty is in the eye of the beholder.’ Beyond this, it appears that beauty provides advantages. Research has found physical attractiveness to be linked to an array of outcomes, such as health [1], perceived intellectual competence [2], reproductive success [3], and general treatment by others [4]. Within the field of criminal justice, attractiveness appears to also be related to offending [5, 6]. If caught, being physically attractive may prove to be advantageous—with research indicating that physically attractive people are treated more leniently by criminal justice actors [7].

### Attractiveness and the criminal justice system

Research to date has generally found that fortune favors the beautiful, with studies finding attractive individuals have better health outcomes [1], as well as increased reproductive success [3]. As it pertains to criminal justice, research suggests that physical attractiveness tends to afford individuals more lenience in the criminal justice system. Beaver et al. [7] examined how physical attractiveness was related to involvement in criminal activity, arrest, conviction, incarceration, and probation. Using data from the National Longitudinal Study of Adolescent to Adult Health, the researchers found that people who were considered attractive were less likely to engage in criminal behavior and less likely to be arrested or convicted. Examining waves 1 through 3 of the same dataset, Mocan and Tekin [5] assessed the relationship between attractiveness and involvement in criminal behaviors including property, drug, and violent crimes. The researchers found participant attractiveness remained relatively stable from early adolescence into early adulthood. Results indicated that even after taking into account a range of familial and personal characteristics, being perceived as very attractive was negatively related to criminal involvement, while being perceived as unattractive was positively related to criminal involvement. Mocan and Tekin [5] also found that the addition of measures related to personality and grooming did not impact the observed relationship.

Some research in this area also finds that there may be a gender effect when assessing these relationships. For example, when examining outcomes by gender, Beaver et al. [7] found that, while the effect sizes were small, there appears to be a leniency effect for attractive females across various criminal justice outcomes. This relationship, however, was not found for males. In their assessment of sentencing outcomes, Ahola et al. [8] found that attractiveness was correlated with perceptions of higher trustworthiness and lower disagreeableness and insensitiveness in females but found no correlation for males. Given these results, the question is why would attractive people be treated more leniently? Although theoretical explanations in this area are limited [9], a potential explanation may be borrowed from research on sentencing and decision-making.

### Conceptual framework

Within sentencing literature, research on judicial decision-making points to the use of conceptual shorthands that are used in the decision-making process. Steffensmeier et al. [10] suggest that judges use three focal concerns—blameworthiness, protection of the community, and practical constraints—to decide how to punish defendants. These focal concerns are generally relied upon because judges often do not have all the pertinent information available to them at the time of sentencing. As such, they rely upon these shorthands to make their sentencing decisions. Research in this area suggests that, in addition to legal factors, judges may take into consideration a variety of extralegal factors—such as race, gender, and socioeconomic status—when making these decisions [10].

For the purposes of the current study, the idea of focal concerns may be applied to the perception of physical attractiveness. As mentioned previously, the way individuals present themselves may influence the way they are perceived. Frazier et al. [11] found that individuals who were well-groomed and nicely dressed were more likely to be granted bond during pretrial proceedings. Interviews with judges corroborated these findings, with judges stating that they “eyeball” defendants and sometimes take their appearance into consideration when making decisions [11].

Steffensmeier et al.’s [10] conceptualization of focal concerns aligns closely with the theory of status characteristics proposed by Berger and colleagues in the 1970s [9, 12, 13] and status generalization theory [2, 14]. Both status characteristics theory and status generalization theory posit that individuals generate beliefs and assumptions of others based on distinct status characteristics, such as gender, race, age, or in this case, attractiveness [9, 13, 15]. These assumptions then govern perceptions of others’ character and expected behavior. Webster and Driskell [9] suggest that attractiveness acts as a diffuse status characteristic, or one that varies by individual and is used as a marker for behavioral expectations even when the characteristic is not directly related to the behavior. In this way, it may be that physically attractive people are perceived as more trustworthy and less capable of committing crimes, and as such, are treated more leniently because of this. Conceptualized further, the phenomenon known as the “halo effect” suggests that attractive people are treated positively because they are perceived as being good or as possessing more socially desirable traits [9, 16–18].

Findings from research on attractiveness and trustworthiness generally support these contentions. Johnson and King [17] used data from the Minnesota Sentencing Guidelines Commission to examine the relationship between multiple appearance characteristics and sentencing decisions. Results indicated that more attractive and “baby-faced” individuals were perceived as less threatening, while those with visible face tattoos and scars were perceived as more threatening. Higher perceived attractiveness and being baby-faced were related to reduced odds of imprisonment, while face tattoos were related to increased odds of imprisonment.

Similarly, Wilson and Rule [19] found people who were deemed as less trustworthy by survey respondents received more severe sentencing decisions. Estrada-Reynolds et al. [20] conducted three studies to assess if masculinity is related to feelings that someone is more violent and more likely to reoffend. The researchers first created standardized computer-morphed images of low-masculine, high-masculine, and moderate-masculine men. They then asked participants recruited through Amazon MTurk to indicate how likely they felt each individual was of committing a crime and how likely they were to reoffend. The three crimes used as variables were assault, burglary, and fraud. In all three studies, men who were perceived as more masculine were significantly more likely to be selected as being more likely to reoffend, as well as to have committed assault or any of the crimes involved in the study.

Taken together, it appears that physically attractive individuals are judged more favorably than their less attractive counterparts. Webster and Driskell [9] note, however, that “…the burden of proof is placed upon demonstrating that status is *not* relevant to ability, instead of the other way around.” As such, an investigation into how other features of personal presentation influence perceptions will add to research surrounding the impact of extralegal factors on criminal justice processing decisions.

## Current study

Research suggests that attractive people are granted more privileges across varying life contexts [2–4, 7]. In regard to the criminal justice system, these individuals appear to be treated more leniently than those who are not deemed attractive. This leniency may be the result of the halo effect, whereby their attractiveness may be construed as an outward representation of their inner good.

Research in this area has generally only used a singular measure of attractiveness [7], without taking into consideration how other aspects of personal presentation may influence how others perceive an individual’s level of attractiveness. This is important to consider because studies have shown that characteristics of personal presentation, like grooming and clothing choice, influence others’ perceptions of various traits such as maturity, trustworthiness, and competence, amongst others [21–23]. Given this, we hypothesize that beyond physical attractiveness, additional factors of personal appearance and presentation may influence how criminal justice actors perceive individuals they encounter. The purpose of the current study is to extend the work done by Beaver et al. [7] by examining how features of personal presentation relate to criminal involvement and a variety of criminal justice outcomes. More specifically, we include measures of grooming and personality to the analysis of attractiveness—as these features may contribute to how individuals are perceived and were not included in Beaver et al.’s [7] assessment of attractiveness. Additionally, the current study builds upon the work of Mocan and Tekin [5] by examining how these measures of personal presentation impact not only criminal behavior, but also various criminal justice processing outcomes.

It is important to note that the evaluations of attractiveness, personality, and grooming used in the current study are derived from interviewer responses of respondent attractiveness, a common practice in studies of attractiveness [1]. As it is said that beauty is in the eye of the beholder, there may be questions regarding the reliability of such judgements given potential variation of opinion across individuals. A meta-analysis conducted by Langlois et al. [4] identified significant interrater reliability estimates regardless of age, ethnicity, or culture. These results indicate that, regardless of context, raters agree on judgements of attractiveness. Additionally, these findings corroborated those found by Feingold [16], whose meta-analysis identified a significant reliability estimate (*r* = 0.83) among studies of Canadian and American citizens published between 1983 and 1989. Finally, Mocan and Tekin [5] also used the Add Health data and found agreement between interviewers on judgements of respondent attractiveness. Additionally, assessment of attractiveness, personality, and grooming measures across all four waves reveal a cross-wave correlation at the .01 level, suggesting stability in attractiveness, personality, and grooming measures across waves. Principal components analyses were also performed for physical attractiveness, personality, and grooming scales. This analysis suggested the four items hung together on a single component with an eigenvalue greater than 1.00 and the next closest eigenvalue being below 1.00 for each scale suggesting that the indicators loaded onto a single latent construct.

## Materials and methods

### Data

Data for the current study were obtained from the National Longitudinal Study of Adolescent to Adult Health (Add Health) [24]. Add Health is a nationally representative sample of adolescents in grades 7 through 12 during the 1994 school year. Add Health is a five-wave longitudinal study conducted both in school and at home. Sampling began at the school-level and the initial sampling frame consisted of 26,666 schools. Prior to sampling, schools were stratified by size, type, region, urbanization, and percent white. Each student attending the 132 schools was asked to complete a 45-minute self-report questionnaire about their school life, friends, background, parent’s background, schoolwork and activities, general health status, and health-related behaviors—resulting in a sample of 90,118 students.

The current study draws on four data files: (1) the wave 1 in-home questionnaire (respondents in grades 7–12), (2) the wave 2 in-home questionnaire (respondents in grades 8–12), (3) the wave 3 in-home questionnaire (respondents aged 18 to 26), and (4) the wave 4 in-home questionnaire (respondents aged 24 to 32). The current study utilizes the Add Health’s restricted use data files, which contains information from over 20,000 individuals. Due to oversampling, some individuals had a higher probability of being included in the sample than others (i.e., not randomly selected). For this reason, survey weights for the corresponding wave will be used in all models. The survey weights used in the current study are provided by the Add Health researchers [25]. Written informed consent was obtained from all Add Health participants for participation in all Add Health aspects. Project protocols were approved by the University of North Carolina School of Public Health Institutional Review Board.

### Measures

#### Dependent variables

*Criminal behavior*. The criminal behavior scale was taken from the wave 4 in-home interviews with the respondent. To create this variable, a scale measuring the respondent’s criminal behavior was generated. Respondents were asked how many times in the last 12 months they: damaged property that did not belong to them; stole something worth less than 50 dollars; stole something worth more than 50 dollars; went into a house or building to steal something; used or threatened to use a weapon to get something from somebody; sold drugs such as marijuana; participated in a physical fight with a group of their friends; bought, sold, or held stolen property; used another individual’s credit card, bank card, or automatic teller card without their permission or knowledge; deliberately wrote a bad check; got into a serious fight; or hurt someone badly enough in a fight that the individual needed care from a doctor or nurse. Possible responses were 0 = *never*, 1 = *1 or 2 times*, 2 = *3 or 4 times*, 3 = *5 or more times*. For the current study, all variables were coded dichotomously such that 0 = *none* and 1 = *1 or more*. In order to create a criminal behavior scale, all items were summed together, with higher values being indicative of more criminal behavior (α = 0.70). A similar scale was used by Beaver et al. [7].

*Arrest*. Arrest was measured during wave 4 using one question. Specifically, the question asked, “Have you ever been arrested?” Answers were coded *0 = No* and *1 = Yes*.

*Probation*. During wave 4, respondents were asked, “Have you ever been on probation for an offense?” Responses were coded dichotomously so that 0 = *No* and 1 = *Yes*.

*Criminal conviction*. During wave 4, respondents were asked, “Have you ever been convicted of or pled guilty to any charges other than a minor traffic violation?” Responses were coded dichotomously so that 0 = *No* and 1 = *Yes*.

*Incarceration*. During wave 4, respondents were asked, “Have you ever spent time in a jail, prison, juvenile detention center or other correctional facility?” Responses were coded such that 0 = *No* and 1 = *Yes*. Importantly, any interviews that were conducted while the respondents were in prison were also coded as being arrested, convicted, and incarcerated.

#### Key independent variables

*Physically attractive*. During waves 1 through 4, interviewers were asked to indicate the respondents’ level of physical attractiveness. Responses were coded such that 1 = *very unattractive*, 2 = *unattractive*, 3 = *about average*, 4 = *attractive*, 5 = *very attractive*. A Physical Attractiveness scale was then created by averaging the interviewer’s responses across all four waves, with higher values indicating higher attractiveness. This exact scale has been used in previous studies using the Add Health [1, 5, 7].

*Attractive personality*. During waves 1 through 4, interviewers were asked to indicate how attractive the respondents’ personality was. Responses were coded such that 1 = *very unattractive*, 2 = *unattractive*, 3 = *about average*, 4 = *attractive*, 5 = *very attractive*. A Personality Attractiveness scale was then created by averaging the interviewer’s responses across all four waves, with higher values indicating higher personality attractiveness.

*Well-groomed*. During waves 1 through 4, interviewers were asked to indicate how well-groomed each respondent was. Responses were coded such that 1 = *very poorly groomed*, 2 = *poorly groomed*, 3 = *about average*, 4 = *well-groomed*, 5 = *very well-groomed*. A Grooming scale was then created by averaging the interviewer’s responses across all four waves, with higher values indicating higher levels of grooming.

#### Control variables

*Age*. A count variable measured at wave 4 indexed the respondent’s age. Responses ranged from 24 to 34.

*Male*. A dichotomous variable measured at wave 1indexed the participant’s sex (0 = *female*, 1 = *male*).

*Black*. A dummy variable measured at wave 1 indexed the participant’s race (0 = *non-Black*, 1 = *Black*).

*White*. A binary variable measured at wave 1 indexed the respondent’s race (0 = *non-White*, 1 = *White*).

*Hispanic*. A binary variable measured at wave 1 indexed the respondent’s ethnicity (0 = *non-Hispanic*, 1 = *Hispanic*). Descriptive statistics for all variables and scales used in the analysis can be found in Table 1.

**Table 1:** Descriptive statistics.

	Mean	SD	Min.	Max.	n
*Dependent Variables*					
Criminal Behavior	0.31	0.92	0	12	15,635
Arrested	0.29	0.45	0	1	15,648
Probation	0.14	0.34	0	1	15,674
Convicted	0.13	0.33	0	1	15,671
Incarcerated	0.16	0.36	0	1	15,680
*Independent Variables*					
Attractiveness	3.52	0.59	1	5	20,768
Personality	3.59	0.57	1	5	20,771
Well-groomed	3.50	0.54	1	5	20,772
*Control Variables*					
Age	28.53	1.78	24	34	15,701
Black	0.23	0.42	0	1	20,704
White	0.62	0.47	0	1	20,704
Hispanic	0.17	0.38	0	1	20,704
Male	0.49	0.50	0	1	20,743

Note: SE = Standard Deviation.

### Analytic plan

The analysis proceeded in a series of four key steps. First, the relationship between physical attractiveness and the key dependent criminal justice variables was examined, while controlling for multiple demographic variables. Second, the analysis was re-estimated, with the inclusion of both the personality and grooming scales, to allow for the assessment of the relationship between each appearance/presentation measure and the criminal justice outcomes while controlling for the others. Following the work of Mocan and Tekin [5] and Beaver et al. [7], who both found gender differences in the relationship between attractiveness and CJ outcomes, we also conducted a gendered analysis. The third step is the full analysis which was estimated for males exclusively. Fourth, the full analysis was estimated for females exclusively.

Given that the key dependent variables of the study are measured continuously (i.e., criminal behavior) and dichotomously (i.e., arrest, probation, criminal conviction, and incarceration), negative binomial and logistic regression, respectively, were utilized. As the measure of criminal behavior is skewed count data, negative binomial regression was used to account for overdispersion. This method was chosen over Poisson regression due to the conditional variance of the measure being larger than the conditional mean of the measure [26]. Results from negative binomial regression are provided through incident risk ratios (*IRR*s), which are calculated by converting and exponentiating the coefficient estimates of the model. The calculation for *IRR* is written as such: *IRR = e* ^*ßtk*^, where *ß*_*tk*_ represents the estimated relationship between covariate *k* and *Y* at time *t*. The resulting *IRR* then describes the percentage change in the rate of criminal behavior as a function of a one-unit change in the independent variable (i.e., physical attractiveness, personality attractiveness, or grooming). An *IRR* above 1.00 indicates a positive association, an *IRR* of 1.00 indicates no association, and an *IRR* below 1.00 indicates a negative association.

The relationship between the attractiveness measures and the criminal justice processing outcomes were analyzed using logistic regression. The estimates are presented using odds ratios (*OR*), which allows for the assessment of outcome likelihood. Similar to *IRR*s, an *OR* above 1.00 indicates a positive association, an *OR* of 1.00 indicates no association, and an *OR* below 1.00 indicates a negative association.

Due to deliberate oversampling during Wave 1 of Add Health data collection, survey weights were used in all analyses. Additionally, a missing variable check was conducted to examine the pattern of missingness across observations. There were no systematic missings. Of the few missings that were observed, most came from the measures of criminal behavior. Because of this, listwise deletion was used, which resulted in 6,155 respondents being removed from the analyses for missing on one or more variables. Importantly, utilizing this method left a large sample size with sufficient statistical power [27]. All statistical analyses were conducted using Stata.

## Results

Table 2 shows the association between physical attractiveness and criminal justice outcomes. Physical attractiveness is significantly and negatively related to all criminal justice variables except probation. In particular, physical attractiveness appears to be related to a reduction in the rate of criminal behavior by 18 percent (*IRR* = 0.82, *p* < .05), reduction in the odds of being arrested by 11 percent (*OR* = 0.89, *p* < .05), conviction by 14 percent (*OR* = 0.86, *p* < .05), and incarceration by 28 percent (*OR* = 0.72, *p* < .05). Age was related to a reduction in the rate of criminal behavior by 11 percent (*IRR* = 0.89, *p* < .05). Being male was related to a significant increase in experiencing all of the measured criminal justice outcomes, with estimates ranging from a 170 percent increase in the rate of criminal behavior (*IRR* = 2.70, *p* < .05) to a 332 percent increase in the odds of receiving probation (*OR* = 4.32, *p* < .05).

**Table 2:** The impact of physical attractiveness on criminal justice outcomes.

	Criminal Behavior		Arrested		Probation		Convicted		Incarcerated	
	**IRR**	**SE**	**OR**	**SE**	**OR**	**SE**	**OR**	**SE**	**OR**	**SE**
Attractiveness	0.82*	(0.05)	0.89*	(0.05)	0.88	(0.06)	0.86*	(0.06)	0.72*	(0.05)
Age	0.89*	(0.02)	0.97	(0.02)	0.96	(0.16)	0.96	(0.02)	0.98	(0.03)
Black	1.61*	(0.19)	1.71*	(0.24)	1.49*	(0.27)	1.67*	(0.32)	1.57*	(0.23)
White	1.02	(0.14)	1.07	(0.13)	1.16	(0.16)	1.31*	(0.18)	0.94	(0.13)
Hispanic	1.26*	(0.12)	1.08	(0.11)	1.08	(0.13)	1.01	(0.15)	1.31*	(0.15)
Male	2.70*	(0.15)	3.59*	(0.18)	4.32*	(0.33)	4.22*	(0.31)	3.73*	(0.29)
	*n* = 14,677		*n* = 14,688		*n* = 14,710		*n* = 14,708		*n* = 14,715	

*p < .05; Note: IRR = Incidence Risk Ratio; OR = Odds Ratio; SE = Linearized Standard Error

Table 3 displays the association between physical attractiveness, personality attractiveness, grooming, and criminal justice outcomes. Here we see that now physical attractiveness was related to an increase in the odds of criminal justice processing when measures of personality attractiveness and grooming are included in the model. Results indicate that physical attractiveness was associated with an increase in the odds of arrest by 63 percent (*OR* = 1.63, *p* < .05), probation by 56 percent (*OR* = 1.56, *p* < .05), conviction by 50 percent (*OR* = 1.50, *p* < .05), and incarceration by 47 percent (*OR* = 1.47, *p* < .05) when taking into account personality and grooming. Conversely, having a more attractive personality was related to a reduction in the odds of arrest by 26 percent (*OR* = 0.74, *p* < .05), probation by 29 percent (*OR* = 0.71, *p* < .05), conviction by 21 percent (*OR* = 0.79, *p* < .05), and incarceration by 38 percent (*OR* = 0.62, *p* < .05). Like personality, grooming is also negatively related to criminal justice outcomes. Respondents who were perceived as being well-groomed experienced a 34 percent reduction in the rate of criminal behavior (*IRR* = 0.66, *p* < .05). Well-groomed respondents were also 55 percent less likely to be arrested (*OR* = 0.45, *p* < .05), 53 percent less likely to be placed on probation (*OR* = 0.47, *p* < .05), 54 percent less likely to be convicted (*OR* = 0.46, *p* < .05), and 57 percent less likely to be incarcerated (*OR* = 0.43, *p* < .05). Age was associated with a 12 percent reduction in the rate of criminal behavior (*IRR* = 0.88, *p* < .05). Being male was related to a significant increase in experiencing all of the measured criminal justice outcomes, with estimates ranging from a 162 percent increase in the rate of criminal behavior (*IRR* = 2.62, *p* < .05) to a 308 percent increase in the odds of receiving probation (*OR* = 4.08, *p* < .05).

**Table 3:** The impact of attractiveness, personality, and grooming on criminal justice outcomes.

	Criminal Behavior		Arrested		Probation		Convicted		Incarcerated	
	IRR	SE	OR	SE	OR	SE	OR	SE	OR	SE
Attractiveness	1.05	(0.09)	1.63*	(0.12)	1.56*	(0.15)	1.50*	(0.14)	1.47*	(0.12)
Personality	0.95	(0.07)	0.74*	(0.06)	0.71*	(0.07)	0.79*	(0.07)	0.62*	(0.05)
Well-groomed	0.66*	(0.07)	0.45*	(0.04)	0.47*	(0.04)	0.46*	(0.04)	0.43*	(0.04)
Age	0.89*	(0.02)	0.97	(0.02)	0.96	(0.02)	0.95	(0.03)	0.98	(0.02)
Black	1.59*	(0.19)	1.62*	(0.23)	1.40	(0.25)	1.58*	(0.30)	1.45*	(0.21)
White	1.00	(0.11)	1.02	(0.13)	1.11	(0.16)	1.25	(0.17)	0.87	(0.12)
Hispanic	1.26*	(0.13)	1.05	(0.10)	1.05	(0.13)	0.98	(0.14)	1.26*	(0.14)
Male	2.62*	(0.14)	3.44*	(0.18)	4.08*	(0.31)	4.00*	(0.30)	3.50*	(0.26)
	*n* = 14,677		*n* = 14,688		*n* = 14,710		*n* = 14,708		*n* = 14,715	

*p < .05; Note: IRR = Incidence Risk Ratio; OR = Odds Ratio; SE = Linearized Standard Error

Table 4 displays the relationship between physical attractiveness, personality attractiveness, grooming, and criminal justice outcomes for males only. Physically attractive males experienced a 60 percent increase in the odds of being arrested (*OR* = 1.60, *p* < .05), a 47 percent increase in the odds of receiving probation (*OR* = 1.47, *p* < .05), a 51 percent increase in the odds of being convicted (*OR* = 1.51, *p* < .05), and a 45 percent increase in the odds of being incarcerated (*OR* = 1.45, *p* < .05). Personality was once again related to a decrease in the odds of experiencing criminal justice outcomes. Males with attractive personalities had 22 percent lower odds of being arrested (*OR* = 0. 78, *p* < .05), 28 percent lower odds of being placed on probation (*OR* = 0.72, *p* < .05), 23 percent lower odds of being convicted (*OR* = 0.77, *p* < .05), and 39 percent lower odds of being incarcerated (*OR* = 0.61, *p* < .05). Similarly, well-groomed males had 33 percent lower rates of criminal behavior (*IRR* = 0.67, *p* < .05), 52 percent lower odds of being arrested (*OR* = 0.48, *p* < .05), 46 percent lower odds of being placed on probation (*OR* = 0.54 *p* < 0.5), 49 percent lower odds of being convicted (*OR* = 0.51, *p* < .05), and 52 percent lower odds of being incarcerated (*OR* = 0.48, *p* < .05). Older males also experienced a 12 percent reduction in the rate of criminal behavior (*IRR* = 0.88, *p* < .05).

**Table 4:** The impact of attractiveness, personality, and grooming on criminal justice outcomes for males.

	Criminal Behavior		Arrested		Probation		Convicted		Incarcerated	
	IRR	SE	OR	SE	OR	SE	OR	SE	OR	SE
Attractiveness	0.99	(0.11)	1.60*	(0.16)	1.47*	(0.16)	1.51*	(0.17)	1.45*	(0.14)
Personality	1.00	(0.11)	0.78*	(0.08)	0.72*	(0.08)	0.77*	(0.08)	0.61*	(0.06)
Well-groomed	0.67*	(0.10)	0.48*	(0.05)	0.54*	(0.06)	0.51*	(0.06)	0.48*	(0.05)
Age	0.88*	(0.02)	0.98	(0.02)	0.96	(0.03)	0.96	(0.02)	0.99	(0.03)
Black	1.54*	(0.21)	1.65*	(0.27)	1.42	(0.30)	1.71*	(0.34)	1.52*	(0.25)
White	1.03	(0.13)	1.02	(0.15)	1.02	(0.16)	1.29	(0.18)	0.87	(0.14)
Hispanic	1.21	(0.13)	1.15	(0.13)	0.98	(0.14)	1.08	(0.16)	1.36*	(0.18)
	*n* = 6,871		*n* = 6,882		*n* = 6,894		*n* = 6,891		*n* = 6,896	

*p < .05; Note: IRR = Incidence Risk Ratio; OR = Odds Ratio; SE = Linearized Standard Error

Finally, Table 5 shows the relationship between physical attractiveness, personality attractiveness, grooming and criminal justice outcomes for females only. Similar to the results for males, physical attractiveness was related to an increase in the risk for experiencing criminal justice outcomes—when controlling for personality and grooming—while personality attractiveness and grooming was associated with a decrease in the risk for these outcomes. In particular, being perceived as physically attractive is related to a 70 percent increase in the odds of being arrested for female respondents (*OR* = 1.70, *p* < .05). Females are 85 percent more likely to be placed on probation (*OR* = 1.85, *p* < .05), 47 percent more likely to be convicted (*OR* = 1.47, *p* < .05), and 56 percent more likely to be incarcerated (*OR* = 1.56, *p* < .05) if they are deemed physically attractive. Having an attractive personality was related to a reduction in the odds of arrest by 31 percent (*OR* = 0.69, *p* < .05), reduction in the odds of receiving probation by 30 percent (*OR* = 0.70, *p* < .05), and reduction in the odds of incarceration by 33 percent (*OR* = 0.67, *p* < .05). Being well-groomed was associated with a reduction in the rate of criminal behavior by 36 percent (*IRR* = 0.64, *p* < .05). Further, well-groomed females have 60 percent lower odds of being arrested (*OR* = 0.40, *p* < .05), 63 percent lower odds of receiving probation (*OR* = 0.37, *p* < .05), 65 percent lower odds of being convicted (*OR* = 0.35, *p* < .05), and 68 percent lower odds of being incarcerated (*OR* = 0.32, *p* < .05). Age was also related to criminal justice outcomes, with older females having 11 percent lower rates of criminal behavior (*IRR* = 0.89, *p* < .05) and 5 percent lower odds of being arrested (*OR* = 0.95, *p* < .05).

**Table 5:** The impact of attractiveness, personality, and grooming on criminal justice outcomes for females.

	Criminal Behavior		Arrested		Probation		Convicted		Incarcerated	
	IRR	SE	OR	SE	OR	SE	OR	SE	OR	SE
Attractiveness	1.16	(0.15)	1.70*	(0.16)	1.85*	(0.26)	1.47*	(0.22)	1.56*	(0.22)
Personality	0.87	(0.11)	0.69*	(0.07)	0.70*	(0.10)	0.86	(0.14)	0.67*	(0.09)
Well-groomed	0.64*	(0.07)	0.40*	(0.05)	0.37*	(0.06)	0.35*	(0.06)	0.32*	(0.06)
Age	0.89*	(0.02)	0.95*	(0.02)	0.97	(0.03)	0.96	(0.04)	0.98	(0.03)
Black	1.70*	(0.36)	1.59*	(0.29)	1.35	(0.36)	1.22	(0.35)	1.28	(0.33)
White	0.96	(0.19)	1.05	(0.17)	1.48	(0.32)	1.15	(0.30)	0.90	(0.20)
Hispanic	1.35	(0.24)	0.88	(0.11)	1.22	(0.22)	0.71	(0.19)	1.03	(0.19)
	*n* = 7,806		*n* = 7,806		*n* = 7,816		*n* = 7,817		*n* = 7,819	

*p < .05; Note: IRR = Incidence Risk Ratio; OR = Odds Ratio; SE = Linearized Standard Error

## Discussion

Previous research has found that physical attractiveness is related to a host of benefits such as reproductive success [3], health outcomes [1], and treatment by others [4]. Research on this subject tends to find that physical attractiveness affords individuals benefits and advantages. Some studies, however, neglect the inclusion of other measures that may be related to attractiveness, such as personality and general physical presentation. The current study sought to expand this line of inquiry by examining how multiple facets of personal appearance and presentation—physical attractiveness, personality, and grooming—are related to various criminal justice outcomes.

Findings revealed that before looking at personality and grooming, physical attractiveness was related to a decrease in criminal behavior and significantly reduced the odds of being arrested by 11 percent, the odds of being convicted by 14 percent, and the odds of being incarcerated by 28 percent—controlling for race, age, and gender. Once measures of personality and grooming were included, however, physical attractiveness was related to a significant *increase* in the odds of arrest (63%), probation (56%), conviction (50%), and incarceration (47%). Further, having an attractive personality reduced the odds of arrest, probation, conviction, and incarceration—controlling for physical attractiveness, race, age, and gender. Being well-groomed was associated with a significant decrease in the rate of criminal behavior by 34 percent, odds of arrest by 55 percent, odds of probation by 53 percent, odds of conviction by 54 percent, and odds of incarceration by 57 percent. These findings are contradictory to prior studies [7] and highlight the importance of considering other factors of appearance and personal presentation when considering how attractiveness influences criminal justice outcomes.

Findings also indicated that when splitting the sample by gender, the results remained relatively similar for males and females. For both males and females, attractiveness was related to an increase in the risk of negative criminal justice outcomes, while having an attractive personality and being well-groomed was associated with a reduction in the risk of experiencing these outcomes. Finally, males were more likely to be arrested, convicted, and incarcerated—controlling for physical attractiveness, personality attractiveness, and grooming.

While speculative, we do propose a few potential explanations for these findings. As mentioned previously, personal presentation is multifaceted and impacts the way in which individuals are perceived. Regarding the results found here, it may be that personality and grooming reduce the risk of experiencing negative criminal justice outcomes because these characteristics may be perceived as requiring more effort to display than physical attractiveness alone. Put differently, it may be, as Wong and Penner [28] suggest, “…*being* attractive is not enough; it is *doing* attractiveness appropriately…” that matters. This may lead criminal justice actors to perceive individuals who put in this effort as more trustworthy or more concerned with appearing as upstanding, as is suggested by the halo effect. In accordance with status characteristics and status generalization theories, these perceptions may subsequently result in more lenient treatment of those individuals.

Alternatively, it may be that due to the correlation between grooming, personality, and attractiveness, the inclusion of personality and grooming in the model reveals a more accurate relationship between attractiveness and criminal justice outcomes. Recall that attractiveness was related to a decrease in all criminal justice outcomes when it was the only measure of personal presentation included in the model. When personality and grooming were controlled for, however, attractiveness was rendered non-significant in its relationship with engaging in criminal behavior but became positively related to all observed criminal justice processing outcomes. Addressing first the non-significant impact of attractiveness on criminal behavior when personality and grooming were included in the model, it may be that personality in particular accounts for the change in outcome. The measure of criminal behavior used in the current study is based on respondent self-reports of behavior and as such is a measure that does not rely on the perceptions of others for the observed outcomes. Therefore, it is possible that having an attractive personality is more strongly related to criminal behavior, reducing the impact of attractiveness.

We turn now to the observed inversion of the relationship between attractiveness and criminal justice processing outcomes after the inclusion of personality and grooming measures. In these outcomes, it is possible that both personality and grooming are more salient when assessing processing outcomes because these outcomes are based on perceptions by others. In other words, having a nice personality and being well-groomed may reduce punishment severity because they present the individual as being pleasant, easy to work with, and/or as someone who has put in the effort to present as such. While these act as protective factors, simply being attractive may then be perceived as a challenge to the individual’s preconceived status generalization of what is good, resulting in harsher punishment. It is important to note that, once again, these explanations are speculative and highlight the need for further investigation in this area.

Although these findings present new evidence for the relationship between attractiveness and criminal justice outcomes, there remain a few limitations we must address. First, perceptions of physical attractiveness, personality, and grooming are subjective and as Beaver et al. [7] indicate, the measure used here relies on the perception of one interviewer across multiple waves. This measure, however, does use an average of the interviewer rating across the four waves and therefore should be more reliable than a single measure [7]. Additionally, research suggests that judgements of attractiveness tend to be fairly universal across contexts [4, 16]. While the measures here come from study interviewers’ perceptions, it is likely that law enforcement, judges, and probation officers are generally making the same judgements about attractiveness, grooming, and personality since—much like the Add Health interviewers—they are making the conclusions with only a short interaction with the individual. Second, the sample for the current study comes from the Add Health, which is a US-based study. As such, the results found here may differ if assessed within a different national and cultural context [7, 29]. Third, the measure of criminal behavior used here does not distinguish by crime type, such as personal, property, and/or violent crimes. The use of a single “catch all” measure of criminal behavior was done to better gauge the impact of the key independent variables on any form of criminal behavior. Although beyond the scope of the current study, future research should investigate the potential for differential impacts of attractiveness, personality, and grooming based on crime categories. There are also a limited number of control variables included in the analyses. As the current study sought to extend the work conducted by Beaver and colleagues [7], we chose to use the same control variables used in their study. Future research should seek to include additional relevant covariates to investigate potential outside influences. In particular, items such as prior record and offense severity may alter the observed outcomes.

It could also be argued that because a large number of models were analyzed, the possibility of multiple testing bias exists (i.e., the possibility of identifying a spurious association is increased). One way to account for this is to use a Bonferroni correction to the p-value [30]. When utilizing this method in the current study, there are a few notable findings. In Table 2, the relationship between attractiveness and arrest (*b* = -0.12, uncorrected *p* = 0.04) and attractiveness and conviction (*b* = -0.15, uncorrected *p* = 0.03) are no longer significant. In Table 3, the association between an attractive personality and conviction is no longer significant (*b* = -0.24, uncorrected *p* = 0.01). Turning to Table 4, the relationship between an attractive personality and arrest (*b* = -0.25, uncorrected *p* = 0.013), attractive personality and probation (*b* = -0.33, uncorrected *p* = 0.005), and attractive personality and conviction (*b* = -0.27, uncorrected *p* = 0.014) are no longer significant. Additionally, the association between personal grooming and criminal behavior (*b* = -0.39, uncorrected *p* = 0.007) was no longer significant. Looking at Table 5, the relationship between attractiveness and conviction (*b* = 0.39, uncorrected *p* = 0.01) is no longer significant, the association between having an attractive personality and probation (*b* = -0.36, uncorrected *p* = 0.015) as well as attractive personality and incarceration (*b* = -0.40, uncorrected *p* = 0.004) are no longer significant. As noted by VanderWeele and Mathur [30], these findings should be interpreted with caution as some have argued that it is an overcorrection and is too stringent.

Despite these limitations, the current study extends the prior knowledge on the relationship between physical attractiveness and criminal justice processing outcomes. In short, we find that when examined alone, physical attractiveness is advantageous when coming into contact with the criminal justice system. When considered alongside personality attractiveness and grooming, however, physical attractiveness becomes disadvantageous, with attractiveness appearing to increase risk of experiencing negative criminal justice outcomes while having an attractive personality and being well-groomed reduce this risk. The results found here point to a need to further reassess how attractiveness relates to a variety of life outcomes.
